# Continuous Distant Measurement of the User’s Heart Rate in Human-Computer Interaction Applications

**DOI:** 10.3390/s19194205

**Published:** 2019-09-27

**Authors:** Jaromir Przybyło

**Affiliations:** AGH University of Science and Technology, 30 Mickiewicza Ave., 30-059 Krakow, Poland; przybylo@agh.edu.pl

**Keywords:** video pletysmography, image processing, heart rate estimation, human-computer interaction, biomedicine, healthcare, assisted living

## Abstract

In real world scenarios, the task of estimating heart rate (HR) using video plethysmography (VPG) methods is difficult because many factors could contaminate the pulse signal (i.e., a subjects’ movement, illumination changes). This article presents the evaluation of a VPG system designed for continuous monitoring of the user’s heart rate during typical human-computer interaction scenarios. The impact of human activities while working at the computer (i.e., reading and writing text, playing a game) on the accuracy of HR VPG measurements was examined. Three commonly used signal extraction methods were evaluated: green (G), green-red difference (GRD), blind source separation (ICA). A new method based on an excess green (ExG) image representation was proposed. Three algorithms for estimating pulse rate were used: power spectral density (PSD), autoregressive modeling (AR) and time domain analysis (TIME). In summary, depending on the scenario being studied, different combinations of signal extraction methods and the pulse estimation algorithm ensure optimal heart rate detection results. The best results were obtained for the ICA method: average *RMSE* = 6.1 bpm (beats per minute). The proposed ExG signal representation outperforms other methods except ICA (*RMSE* = 11.2 bpm compared to 14.4 bpm for G and 13.0 bmp for GRD). ExG also is the best method in terms of proposed success rate metric (*sRate*).

## 1. Introduction

Photopletysmography (PPG) is a non-invasive, low-cost optical technique used to detect volumetric changes in blood in the peripheral circulation. It has many medical applications, including clinical physiological monitoring: blood oxygen saturation and heart rate (HR) [1], respiration [2]; vascular assessment: arterial disease [3], arterial ageing [4], venous assessment [5], microvascular blood flow and tissue viability [6]; autonomic function: blood pressure and heart rate variability [7], neurology [8], and telehealth applications [9].

The PPG sensor has to be applied directly to the skin, which limits its practicality in situations such as freedom of movement is required [10]. Among the various contactless methods for measuring cardiovascular parameters [11], video plethysmography (VPG) have recently become popular. One of the first approaches was proposed by Verkruysse et al. [12], who showed that plethysmographic signals can be remotely measured from a human face in normal ambient light using a simple digital, consumer level photo camera. The advantages of this approach, compared to standard photopletysmography (PPG) techniques, are that it does not require uncomfortable wearable accessories and allows easy adaptation to different requirements in various applications, such as: monitoring the driver’s vital signs in the automotive industry [13], optimization of training in sport [14] and emotional communication in the field of human-machine interaction [15].

Since then, there has been a rapid development of literature on VPG techniques. A summary of 69 studies related to VPG can be found in [16]. Poh et.al [17,18] introduced a new methodology for non-contact, automatic and motion tolerant cardiac pulse measurements from video images based on blind source separation. They used a basic webcam embedded in a laptop to record videos for analysis. To detect faces in video frames and locate the region of interest (ROI) for each video frame, an automatic face detection algorithm was used.

In [19], the authors proposed a framework that uses face tracking to solve the problem of rigid head movements and use the green background value as a reference to reduce the interference from illumination changes. To reduce the impact of sudden non-rigid facial movements, noisy signal segments are excluded from the analysis. Also, several temporal filters were used to reduce the slow and non-stationary trend of the HR signal.

A complementary method for extracting heart rate from video by analyzing subtle skin color changes due to blood circulation has been proposed in [20]. This algorithm is based on the measurement of subtle head movement caused by Newtonian reaction to the influx of blood inflow with each beat. Thus, the method is effective even when the skin is not visible. A typical procedure for extracting a HR signal from a video frame sequence consists of the following stages [21]: selection and tracking of the region of interest (ROI), pre-processing, extraction and post-processing of the VPG signal, pulse rate estimation. Many different published articles present various improvements of one or several stages. For example, in [22] the author proposed using a new signal extraction method: green-red-difference (GRD) as a robust alternative to G. However, a large proportion of them presents the results of tests carried out under controlled conditions (i.e., lighting, short term monitoring, limited or not natural person movements).

In realistic situations, the task of estimating HR is difficult because many factors can contaminate the pulse signal. For example, the movement of a subject consists of a combination of rigid (head tilts, change of position) and non-rigid movements (facial actions, eye blinking). This can affect pixel values of the face region. Fluctuations in lighting caused by changes in the environment include various forms of noise, such as the blinking of indoor lights or computer screen, a flash of reflected light, and the internal noise of a digital camera.

In this article, we propose a video pulse measurement system designed for continuous monitoring of the user’s heart rate (HR) during typical human-computer interaction (HCI) scenarios, i.e., working at the computer. Since physiological activities and changes are a direct reflection of processes in the central and autonomic nervous systems, these signals can be used in an affective computing scenarios (i.e., recognition of human emotions), Assisted Living or healthcare applications (contactless monitoring of cardiovascular parameters). The contribution of this article is following:

- To our knowledge we are the first to systematically study the impact of human activities during various HCI scenarios (i.e., reading text, playing games) on the accuracy of the HR algorithm,
- As far as we know, we are the first to propose the use of new image representation (excess green ExG), which provides acceptable accuracy and at the same time is much faster to compute than other state of the art methods (i.e., blind-source separation—ICA),
- We used the state-of-the art real-time face detection and tracking algorithm, and evaluated four signal extraction methods (preprocessing), and three different pulse rate estimation algorithms,
- To our knowledge we are the first to propose a method of correcting information delay introduced by the algorithm when comparing results with reference data.

The article has the following structure: the next Section 2 describes the experimental setup as well as the algorithmic details. The results and discussion are presented in Section 3, the paper is summarized in Section 4.

## 2. Materials and Methods

The primary goal of this research was to check the effectiveness of the HR algorithm during typical human-computer interaction (HCI) scenarios. Thus, we evaluated four signal extraction methods and implemented three different HR estimation algorithms. We evaluated the effectiveness of selected algorithms using recorded video sequences of participants performing various HCI tasks. The implementation of the proposed methods can be easily adapted to running in real-time framework, however implementation details are not included in this paper.

### 2.1. Experimental Setup

An experimental setup consisted of a RealSense™ SR300 camera (model Creative BlasterX Senz3D, Intel, Santa Clara, CA, USA) that can provide RGB video streams with the following parameters: resolution up to 1280 × 720 pixels at 60 FPS (frames per second). To focus on assessing the impact of noise factors on the results of HR detection, we used RGB channel with a resolution of 640 × 480 pixels and a frame rate of 60 FPS. The camera was located 0.5 to 0.6 m from the volunteers (depending on the experiment).

Various extrinsic factors affect the reliability of VPG HR measurement [23]. One of the factors is change in lighting conditions. This factor requires special attention when the user works with a computer exposed to variable illumination caused by the content displayed on the monitor. Another factor that can affect the accuracy of the HR measurement is the sudden user’s movements, caused for example by emotions while playing computer games. To estimate the impact of these factors on remote HR measurements, we recorded additional signals using a SimpleLink™ multi-standard SensorTag CC2650 (Texas Instruments, Dallas, TX, USA). It is a low energy Bluetooth device, that includes 10 low-power MEMS sensors of which we used ambient light and motion tracking sensors. The SensorTag was placed on the chest of the subject near the neck and face. To measure the ground truth HR, we used the ECG-based H7 Heart Rate Sensor (Polar Electro OY, Kempele, Finland) connected via Bluetooth).

### 2.2. Region of Interest (ROI) Selection and Tracking

There are many sources of changes in the appearance of the face. They can be categorized [24] into two groups—intrinsic factors related to the physical nature of the face (identity, age, sex, facial expression) and extrinsic factors resulting from the scene and lightning conditions (illumination, viewing geometry, imaging process, occlusion, shading). All these factors make face detection and recognition a difficult task. Therefore, in recent years there have been many approaches to detecting faces in natural conditions. Surveys of those methods are presented in articles [25,26].

A fast and reliable implementation of the face detection algorithm can be found in Dlib C++ library [27]. It is based on Histograms of Oriented Gradients (HoG) algorithm proposed in [28], combined with Max-Margin Object Detection (MMOD) [29] which produces high quality detectors from relatively small amounts of training data.

**Figure 1:**
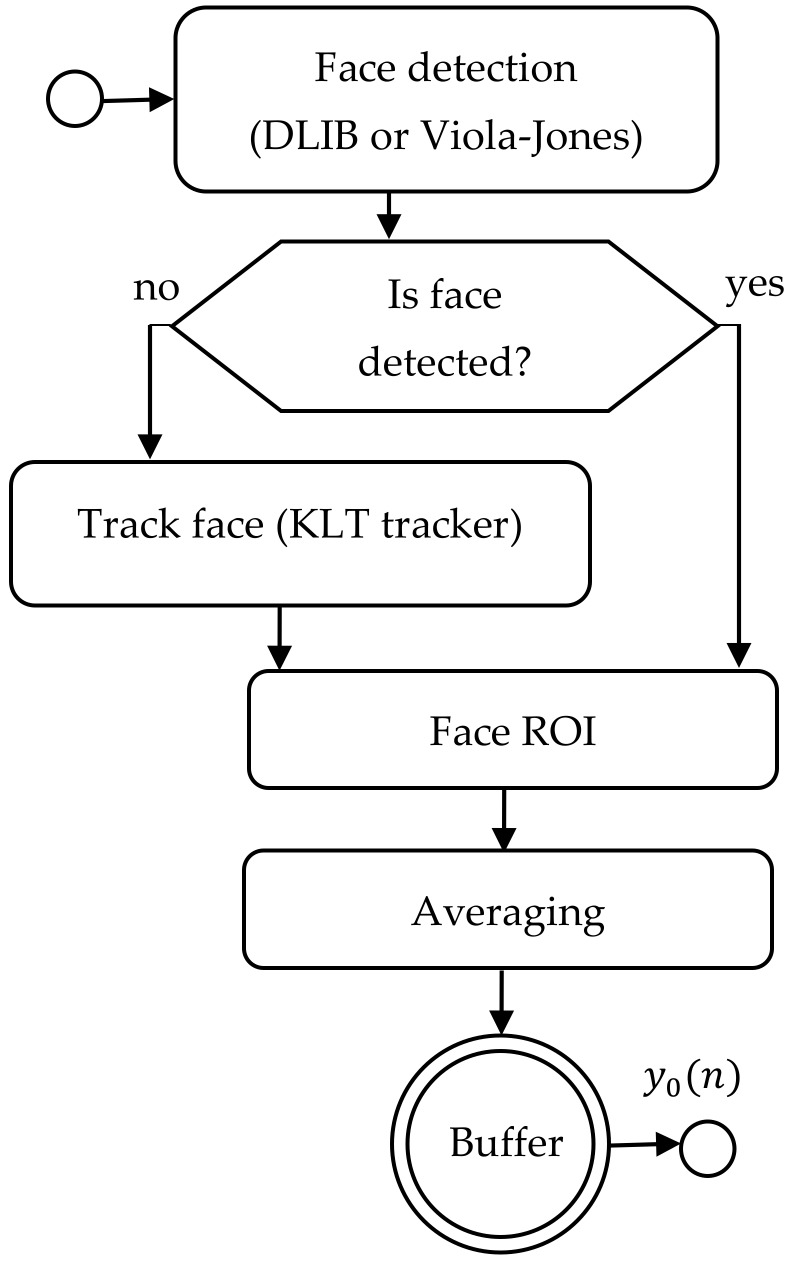
Face detection and tracking—algorithm outline.

In present work, we combined the Dlib’s frontal face detector with the KLT tracking algorithm [30] to effectively follow faces in a video sequence. The outline of the algorithm is presented in Figure 1. The face detector implemented in Dlib library appears to be faster and more robust than the Viola-Jones detector [31]. It shows a low ratio of false positive results, which is essential assumption of our system. However, one of the limitations of this implementation is that the face model was trained using frontal images with the face size at least of 80 × 80 pixels. This means that finding smaller faces requires up-sampling the image (which increases processing time) or re-training the model. Detection of non-frontal faces also requires a different model.

The face detector is applied in each of the consecutive image frames. The resulting bounding box is then used by a heart rate estimation algorithm. If the face is not detected by the Dlib detector, the KLT tracking algorithm is used to track a set of feature points from the previous frame and estimate correct bounding box on the current frame. A feature points (corners) are detected inside the face rectangle using the minimum eigenvalue algorithm [32]. The use of a tracking algorithm minimizes the impact of rigid head movements typical in human-computer interaction scenarios. In case of the Dlib detector fails to detect a face, the system automatically switches to the Viola-Jones detector for a single frame. This allows to correctly reinitialize the tracker.

**Figure 2:**
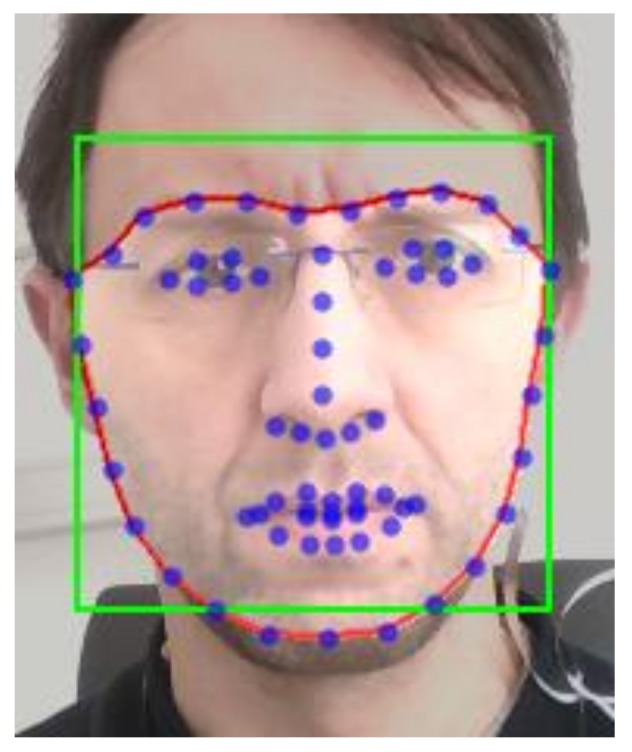
Example image frame with the region of interest (ROI) superimposed.

The calculated bounding box can include not only skin-color pixels (where the pulse signal is expected), but also objects outside the face. To exclude these regions from the HR estimation, a facial landmark detector [33] is used on the cropped part of the image. Based on detected landmark points, a proper region of interest (ROI) is selected for further analysis (Figure 2).

### 2.3. Preprocessing and VPG Signal Extraction

The selected region of interest is then used to calculate the average color intensities over the ROI for each subsequent image frame. These values are stored in a circular buffer of length N, forming the raw VPG signal ***y*_0_**(*n*) = [ R_0_(*n*), G_0_(*n*), B_0_(*n*)]^T^. Then the raw VPG signal is detrended using a simple method consisting of mean-centering and scaling [21] (Equation (1)):

(1) $$y\left(n\right)=\frac{y_{0}\left(n\right)-\mu \left(n,L\right)}{\mu \left(n,L\right)}$$

where $\mu \left(n,L\right)$ is an L-point running mean vector of VPG signal and ***y***(*n*) = [ R(*n*), G(*n*), B(*n*)]^T^.

The strongest VPG signal can be observed in the green (G) channel. Because the camera’s RGB color sensors pick up a mixture of reflected VPG signal along with other sources of fluctuations, such as motion and changes in ambient lighting conditions, various approaches to overcome this problem have been reported in the literature. In [22] a robust alternative to G method has been presented—green-red difference (GRD) which minimizes the impact of artifacts (Equation (2)):

(2) $$GRD\left(n\right)=G\left(n\right)-R\left(n\right)$$

Some authors utilize the fact that each color sensor registers a mixture of original source signals with slightly different weights and uses the independent component analysis (ICA) [17,34]. The ICA model assumes that the observed signals ***y***(*n*) are linear mixtures of sources **s**(*n*). The aim of ICA is to find the separation matrix ***W*** whose output (Equation (3):

(3) $$\hat{s}\left(n\right)=W\cdot y\left(n\right)$$

is an estimate of the vector ***s***(*n*) containing the underlying source signals. The order in which ICA returns the independent components is random. Thus, the component whose power spectrum contained the highest peak can be selected for further analysis. In this work, we used FastICA implementation [35] and calculated power spectrum in the range 35–180 bpm (which corresponds to 0.583–3.00 Hz).

In our research, we found that method for greenness identification [36] utilizing the excess green image component (ExG), amplify the pulse signal and it is faster to compute than the ICA while reducing the impact of noise. The ExG image representation is computed as follows. First, the normalized components r, g and b are calculated using Equation (4):

(4) $$r\left(n\right)=\frac{R\left(n\right)}{R\left(n\right)+G\left(n\right)+B\left(n\right)}\;\;\;\;\;g\left(n\right)=\frac{G\left(n\right)}{R\left(n\right)+G\left(n\right)+B\left(n\right)}\;\;\;\;\;b\left(n\right)=\frac{B\left(n\right)}{R\left(n\right)+G\left(n\right)+B\left(n\right)}$$

The excess green component ExG is defined by Equation (5):

(5) $$ExG\left(n\right)=2\cdot g\left(n\right)-r\left(n\right)-b\left(n\right)$$

**Figure 3:**
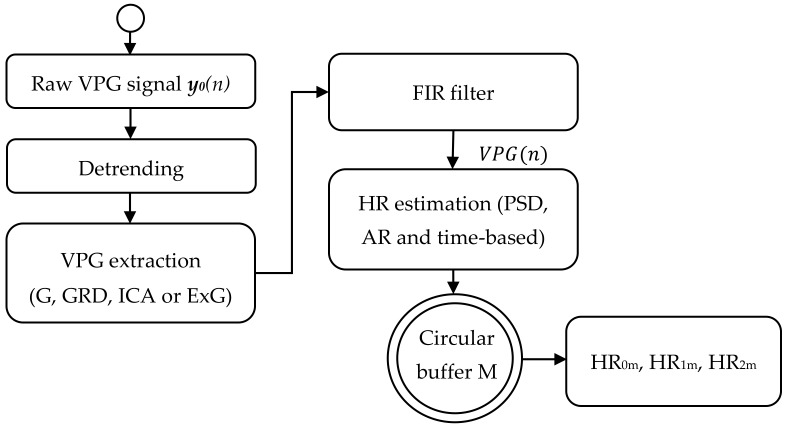
HR estimation algorithm outline.

The refined VPG signal (G, GRD, ICA or ExG) is then band-limited by a zero-phase digital filter (Bartlet-Hamming) yielding the signal VPG(*n*). The summary of the pre-processing, VPG signal extraction and heart rate estimation steps is provided in Figure 3.

### 2.4. Heart Rate Estimation Algorithm

To estimate the heart rate we used three different algorithms. The first algorithm was based on the calculation of the power spectral density (PSD) estimate of the signal VPG(*n*), using the Welch algorithm and the filter bank approach. To find the pulse frequency, the highest frequency peak was located in the PSD, as a result of which the heart rate was estimated (named as HR_0_ in this paper). An important aspect of this classic frequency-based approach is that the frequency resolution *fres* depends on the length of the signal buffer (Equation (6)):

(6) $$f_{res}=\frac{F_{s}}{N}$$

where: N is the length of the signal observation and Fs is the sampling frequency (frame rate of the video).

We also used a second algorithm based on autoregressive (AR) modelling. In the AR model, the input signal can be expressed by Equation (7):

(7) $$y^{\prime }\left(n\right)=-\overset{p}{\underset{k=1}{\sum }}a_{k}\cdot y^{\prime }\left(n-k\right)+e\left(n\right)$$

where: p is the model order, $a_{k}$ are the model coefficients, and $e\left(n\right)$ is the white noise.

Using the Yule-Walker method we fit the AR model to the input signal VPG(*n*) and obtain an estimate of the AR system parameters *ak*. Then, the frequency response of this filter was used to calculate the pulse rate (named as HR_1_ in this paper). The HR_1_ value was estimated by detecting the highest frequency peak in the filter frequency responsein the selected range (50–180 bpm).

The third approach was time-based (depicted as TIME in the article). On the filtered signal VPG(*n*), peaks were located using only the peak detection algorithm. Then the intervals between successive peaks were calculated and their median value was used to obtain the heart rate value (HR_2_).

To minimize false detections, caused by head movements and other sources of image variations, the estimated HR has been further post-processed. A second heart rate buffer of length M was used to store the latest HR_0_, HR_1_ and HR_2_ values. Then the average value of each HR buffer content was calculated and used as a new estimate of the current heart rate (named as HR_0m_, HR_1m_ and HR_2m_ respectively).

### 2.5. Evaluation Methodology

Different kinds of metrics were proposed in other articles for evaluating the accuracy of HR (heart rate) measurement methods. The most common is the root mean squared error denoted as *RMSE* (Equation (8)):

(8) $$\mathrm{RMSE}=\sqrt{\frac{1}{n}\sum_{i=1}^{n}{\left[HR_{error}\left(i\right)\right]}^{2}}$$

(9) $$HR_{error}=HR_{video}-HR_{gt}$$

where: $HR_{video}$– the HR estimated from video, $HR_{gt}$—the ground truth HR values.

Because *RMSE* is sensitive to extreme values or outliers, we additionally propose using a metric that allows to assess how long the accuracy of a given algorithm is within the assumed error tolerance (Equation (10)). This is particularly important in medical applications where measurement reliability is important:

(10) $$\mathrm{sRate}=\frac{100}{n}\cdot \overset{n}{\underset{i=1}{\sum }}\left(\left|HR_{error}\left(i\right)\right|\leq tolerance\right)$$

Little or no attention has been given in literature regarding the effect of information delay introduced by the algorithm on the error metrics. Assuming that the algorithm introduces a delay t_0_ and the measured ground truth HR values are also delayed by t_1_ (due to acquisition and device measurement method), HR_error_ is biased. Therefore, direct comparison of HR values using HR_error_ is not accurate (a systematic error is introduced). In addition, HR_video_ and HR_gt_ usually are sampled at different frequencies. For example, our camera sampling frequency was 60 FPS and the Polar H7 heart rate sensor provides measurements every approximately three seconds.

**Figure 4:**
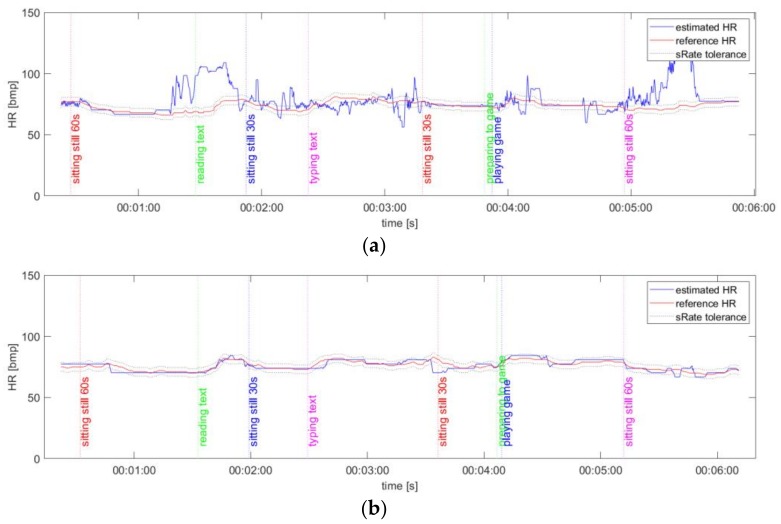
An example of HR time-series plots for algorithm No.1 (PSD) and ExG signal representation: (**a**) video No.5; (**b**) video No.9.

To minimize the impact of delays and different sampling frequencies on the results of the HR comparison, we propose the following method. First, HR_gt_ values are interpolated to match the sampling frequency of HR_video_ using simple linear interpolation, resulting in HR_gt2_. An example of ground truth and measured HR time series is given in Figure 4. All results are available online at [37].

The delay introduced by the algorithm was estimated using the generated artificial signal of known frequency and time of change. Here, we used a signal that changes from 80 to 120 bpm and has a similar amplitude as VPG(*n*). The resulting delays t_0_ do not include the delay t_1_ introduced by the Polar H7 device. We have adopted a constant delay introduced by the measuring device. Assuming that the delay introduced by the algorithm is constant for a given algorithm and its parameters, the estimated delay t_2_ can be used to correctly evaluate the remaining sequences. Although this is a strong assumption, it improves the accuracy of the results. An estimation of the algorithm delay can also be performed using cross-correlation. However, this analysis is not included in the article, because the estimated delays strongly depended on the shape of the signal and the selected fragment. The results are summarized in Table 1.

It is also worth mentioning that delay correction is useful for correctly positioning the beginnings of individual parts of the experiment. For example—the impact of a user’s head movements may be visible only after some time (equal to the algorithm delay) on the estimated pulse signal.

### 2.6. Details of Experiments

The algorithm parameters have been set to:

- Algorithm No. 1 (PSD, Welch’s estimator): the window length N = 1024 samples (which gives a frequency resolution of 3.52 bpm/bin and temporal buffer window of length 21 s),
- Algorithm No. 2 (AR modelling): the order of AR model was equal to 128, the AR model frequency response computed for FFT length of 1024, the window length of N = 600 samples (which gives a frequency resolution of 3.52 bpm/bin and temporal buffer window of length 10 s),
- Algorithm No. 3 (time-based peak detection, depicted as TIME): the buffer length N = 600 samples (which gives a temporal buffer window of length 10 s).

Common parameters for all algorithms were: the bandpass filter of order = 128 and bandwidth = (35–180) bpm (which is equivalent to 0.583–3.00 Hz), the HR postprocessing buffer length M was equivalent to 1 s.

Several video sequences of participants performing HCI tasks were recorded using lossless compression (Huffman codec) and 24-bits-per-pixel format (RGB stream), image resolution of 640 × 480 pixels and frame rate of 60 FPS. Each sequence was approximately 5 min long. The RealSense camera was positioned in such a way that the face of the monitored participant was in the frontal position. All participants were asked to perform various tasks reflecting typical user-computer interaction scenarios. Thus, each video sequence consists of the following parts:

- Part 1—the participant sits still (60 s) without head movements and minimal facial actions,
- Part 2—the participant reads text (short jokes) displayed on the computer screen in front of him, and can express emotions,
- Part 3—the participant sits still (30 s),
- Part 4—the participant rewrites text from the paper located on the left or right side of the desk using the keyboard (which results in head movements),
- Part 5—the participant sits still (30 s),
- Part 6—after the short mental preparation the participant plays the arkanoid game using the mouse and the keyboard,
- Part 7—the participant sits still (60 s).

**Figure 5:**
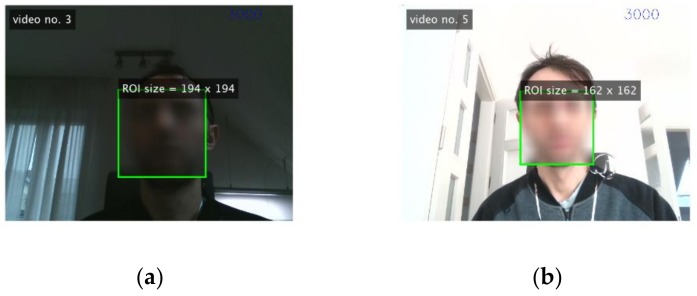
An example of video frames: (**a**) video No.5; (**b**) video No.9.

Only selected parts (1, 2, 4 and 6) were included in the study. The video sequences were recorded in different places and under different conditions (illumination, distance, and if possible similar camera parameters). A description of these videos is provided in Table 2. Examples of video frames are shown in Figure 5. Duration, average illumination values and standard deviation of accelerations for sequences are given in Table A1 and Table A2 (Appendix A).

## 3. Result

### 3.1. Comparison of the VPG Signal Extraction Methods (G, GRD, ICA and ExG)

To select the appropriate statistical methods to compare the results, a Shapiro-Wilk parametric hypothesis test of composite normality can be used. However, with a small sample size (9 videos), the impact of outliers can be significant. Therefore, median and IQR were used as statistical measures.

**Figure 6:**
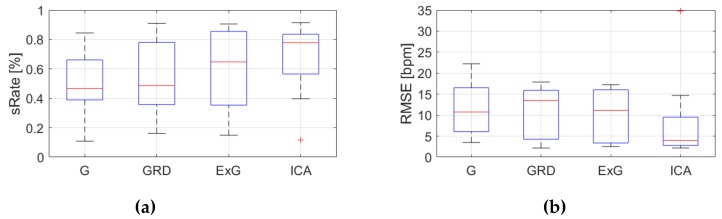
Comparison of signal extraction methods, algorithm No.1 (PSD): (**a**) box plots for *sRate*; (**b**) box plots for *RMSE*. Blue lines—IQR range, red line—median value.

**Figure 7:**
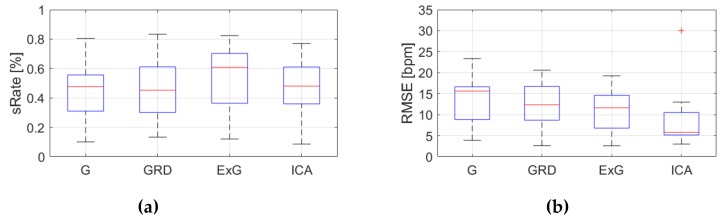
Comparison of signal extraction methods, algorithm No.2 (AR): (**a**) box plots for *sRate*; (**b**) box plots for *RMSE*. Blue lines—IQR range, red line—median value.

**Figure 8:**
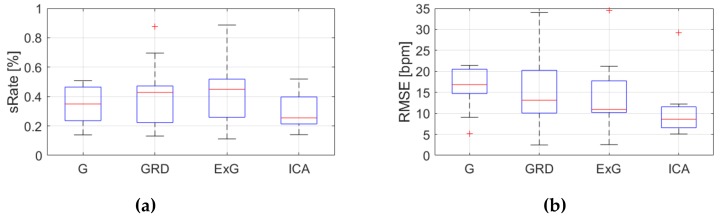
Comparison of signal extraction methods, algorithm No.3 (TIME): (**a**) box plots for *sRate*; (**b**) box plots for *RMSE*. Blue lines—IQR range, red line—median value.

Table A3, Table A4 and Table A5 (Appendix A) show the results of HR estimation for various signal extraction methods and selected algorithms. The results were calculated for entire video sequences (including all participant activities). The *sRate* value is given for a threshold of 3.52 bpm (equal to the algorithm frequency resolution). Box plots (Figure 6, Figure 7 and Figure 8) are also included to better illustrate *sRate* and *RMSE* distributions.

Considering algorithm No. 1 (PSD), the lowest median *RMSE* with low interquartile range (IQR) value is for the ICA signal extraction method. The second lowest *RMSE* values relate to the G and ExG representations. The worst results are for the video No. 9. However, this video was recorded under artificial lighting conditions with lights visible in the scene, which could have a negative effect on the results. Also, the actual heart rate was low (about 50 bpm), which is close to the limit of the measured range (results below 50 bpm are considered incorrect). The *sRate* measure shows similar results—it is the highest for ICA signal extraction method. The ExG method has the highest IQR values.

Looking at the algorithm No. 2 (AR), and *RMSE* - the results are similar to the PSD algorithm. However, all IQR values are lower, which means that this algorithm gives more similar outcome for videos acquired under different conditions. As for *sRate*, the highest value is for ExG signal extraction method but with a large IQR. Given algorithm No. 3 (TIME), the lowest median *RMSE* value with a small interquartile range (IQR) value is for ICA, followed by ExG signal extraction method. All errors are higher for this algorithm than for PSD and AR. The *sRate* is the highest for ExG and then GRD. However, the lowest *sRate* IQR values relate to the ICA and G signal representation.

To compare the medians between groups (signal extraction methods) for statistical differences, a two-sided Wilcoxon rank sum test was used. The Wilcoxon rank sum test is a nonparametric test for the equality of population medians of two independent samples. It is used when the outcome is not normally distributed and the samples are small. The results are shown in Table A6 (Appendix A). The p-values of almost all combinations of signal extraction methods indicate that there is not enough evidence to reject the null hypothesis of equal medians at a default significance level of 5%. This means that all methods provide similar results statistically. The exception is the comparison of G and ICA for algorithm No. 3 (TIME), but only for the *RMSE* metric.

### 3.2. Comparison of the VPG Signal Extraction Methods for Various Activities

To see how individual activities affect the results of heart rate detection, the *RMSE* and *sRate* values of the following video parts have been compared:

- part 1 (the participant sits still for a minimum of 60 seconds),
- part 2 (the participant reads text),
- part 4 (the participant rewrites text using the keyboard and the mouse),
- and part 6 (the participant plays a game).

Because, *RMSE* and *sRate* can be regarded as a small sample size (nine videos) and the effect of outliers can be significant, the median and IQR were used as statistical measures. Figure 9, Figure 10, Figure 11, Figure 12, Figure 13, Figure 14, Figure 15, Figure 16, Figure 17, Figure 18, Figure 19 and Figure 20 show the results of the HR estimation and comparison of the signal extraction methods and selected algorithms for selected parts.

Considering algorithm No. 1 (PSD), *RMSE* and IQR values are lowest for the ICA for parts 1, 2 and 6 (sitting still, reading text and playing game). For the part 4 (rewriting text) the lowest *RMSE* value applies to the ExG signal representation. Given *sRate*, the best representation is ICA for parts 1,2 and 6, but part 4, where the highest *sRate* is for ExG. However, the IQR values are the lowest for ICA only for parts 1 and 6. For parts 2 and 4 the lowest IQR is for G and GRD representations respectively.

The lowest *RMSE* are for parts 1 and 6 (sitting still and playing a game), in which facial actions and head movements were small. Part 2 (reading text) has the highest IQR values. This means that facial actions in some cases have a negative impact on the accuracy of HR estimation. The large head movements present in part 4 (rewriting text) have the least impact on the accuracy of the ExG signal extraction method.

Considering algorithm No.2 (AR), *RMSE* are the lowest for ICA for parts No. 2, 4 and 6 (reading text, rewriting text and playing game). However, IQR values are not always the lowest for ICA. For part 1 (sitting still) the lowest *RMSE* value applies to the ExG representation, but with a high IQR value. Given *sRate*, it is highest for ICA and parts No. 2, 4 (reading and rewriting text). For part No. 6 (playing game) the best signal extraction method is ExG, and for part No.1 (sitting still) the G image representation.

Given algorithm No. 3 (TIME), *RMSE* values are lowest for ICA for all parts. However, *sRate* is highest for the ExG signal extraction method (parts No. 2 and 6) and GRD for part No.1. This means that there are outliers present because *RMSE* is sensitive to extreme values. The IQR of *sRate* is the lowest for G representation and almost all parts.

To compare the medians between groups (signal extraction methods) for statistical differences, a two-sided Wilcoxon rank sum test was used. The results are shown in Table A7, Table A8 and Table A9 (Appendix A). The p-values of almost all combinations of signal extraction methods indicate that there is not enough evidence to reject the null hypothesis of equal medians at a default significance level of 5%. This means that all methods provide similar results for different activities statistically. The exceptions are: comparison between G and ICA for PSD and part 6, G and ICA for AR and part 6 (*RMSE* only), and G and ICA for TIME and parts 1, 4 (*RMSE* only).

### 3.3. Comparison of the Different Algorithms and Activities

The results of comparing different algorithms (PSD, AR, TIME) are shown in Table 3. Statistics were calculated for entire video sequences (including all participant activities).

Considering the median values, the best results (highest *sRate* and lowest *RMSE*) can be observed for algorithm No. 1 based on power spectral density (PSD). The second best algorithm is based on autoregressive modeling (algorithm No. 2). The worst results are for direct analysis of the VPG signal in the time domain (algorithm No. 3). It is worth noting that video No. 9 has a significant impact on results. ICA is the best signal extraction method in terms of *RMSE* values. However, in the case of *sRate* the best results are for ExG.

To compare the medians between groups (algorithms) for statistical differences, a two-sided Wilcoxon rank sum test was used. The results are shown in Table A10 (Appendix A). The p-values of almost all combinations of algorithms and signal extraction methods indicate that there is insufficient evidence to reject the null hypothesis of equal medians at a default significance level of 5%. The only exceptions are: ICA and G for PSD vs TIME, where p-values indicate the rejection of the null hypothesis of equal medians at a default significance level of 5%. This means that the most important issue for the ICA signal extraction method is choosing the right estimation algorithm.

### 3.4. Analysis of the Impact of Average Lighting and User’s Movement on the Results of Pulse Detection.

To assess the effect of the scene illumination on the pulse detection accuracy, a Pearson’s correlation coefficient between the median *sRate* and the average scene lighting was calculated for all video sequences (Table A11 in Appendix A). The results show only one strong positive correlation (0.71) for algorithm No. 3 (TIME) and the GRD signal extraction method. There are no medium and strong correlations present, with a significance level of less than 0.05 for other combination of algorithms and signal extraction methods. This may be due to similar and poor lighting for most video sequences.

Similarly—to assess whether the user’s movements affect the results, correlation coefficients were calculated between the median *sRate* and the standard deviation of the accelerations (measured by SensorTag) for the entire video sequences (Table A12 in Appendix A). The results show strong positive correlations (> 0.6) for:

- algorithm No. 1 (PSD), and GRD, ExG
- algorithm No. 2 (AR), and GRD
- algorithm No. 3, all except ICA

Counterintuitively, *sRate* raises as the standard deviation of the accelerations increases. This might suggest that ballistocardiographic head movements generated by the flow of blood through the carotid arteries has strongest impact than subtle skin color variations caused by circulating blood. Only the ICA image representation is not sensitive to acceleration. It is also worth noting that this might be the effect of the location of the sensor (chest). However, further investigation of this hypothesis is required. Also, the Pearson’s correlation coefficient with a small sample size might lead to inaccurate results. However, it can still provide useful information.

## 4. Discussion

The main purpose of this research was to investigate the impact of human activity on the accuracy of the VPG heart rate algorithm. We focused on activities performed during typical human-computer interaction (HCI) scenarios (i.e., reading text, rewriting text, playing game). Thus, the evaluation of the continuous HR estimation accuracy was carried out on several video sequences recorded in different places and under different conditions (illumination, person identity, distance from the computer screen and camera). We have used state of the art face detection and tracking algorithm, and compare various signal extraction methods, including (to our knowledge) first time used the ExG image representation. It is worth noting that the scene lighting for most of the videos was very poor, which corresponds to the typical computer work conditions.

For the entire video sequence and taking into account the *RMSE* metric, the ICA signal extraction method results in smallest errors. However, when it comes to reliability of measurements and maintaining the accuracy of a given algorithm within the accepted error tolerance (*sRate* metric), the ExG representation seems to be a promising method. This is especially important in medical applications. It is also worth mentioning that the ExG method is much faster to calculate than ICA (about four times—MATLAB implementation on an Intel i7 machine).

To check how individual activities affect the results of heart rate detection, the following activities were compared: the participant sits still for a minimum of 60 seconds, the participant reads text, the participant rewrites text using the keyboard and the mouse, the participant plays game. In conclusion, considering algorithm No.1 (PSD), the ICA signal extraction method works better in sequences where there are no large head movements (sitting still and playing a game). For large head movements, the ExG representation gives better results. Facial actions (part 2 – reading text) have a negative impact on the accuracy of HR estimation. Given algorithm No.2 (AR), it is difficult to indicate the best signal extraction method. In general, ICA works better on parts with facial actions and head movements. For other parts, the ExG method works well, but for part in which the participant was sitting still, the simplest signal representation (G) is the best. Interestingly, these are the opposite results than in the case of the PSD algorithm, in which the ICA signal extraction method works better in cases where there are no large head movements. Considering algorithm No.3 (TIME), the ExG signal representation method provides better reliability of measurements (*sRate*). The smallest *RMSE* is for ICA, but the *RMSE* metric is more sensitive to extreme values and outliers found in the collected data.

Based on the Wilcoxon rank sum test, almost all signal extraction methods provide similar results statistically with the exception of G and ICA comparisons. This means that for the tested videos it is impossible to indicate the best method that works in all scenarios and lighting conditions. Collecting more data can help indicate a better method. Comparing the results obtained from different algorithms, we found that algorithm No. 1 (PSD) gives the best results, followed by the algorithm No. 2 (AR). The accuracy of the algorithm No. 3 (time-based) is significantly different from other algorithms. In addition, based on the Wilcoxon rank sum test, for the ICA signal extraction method the most important is the selection of the appropriate estimation algorithm.

Taking into account individual activities, the highest average *sRate* applies to the activity in which participants sat still. The second highest average *sRate* is for the activity in which users were playing game. The lowest *sRate* value applies to: reading and typing text respectively. Although, the ICA method seems to provide better results, this is not always the case. There are several combinations of estimation algorithm and signal extraction method in which the ExG is better (i.e., part No.1 and TIME).

The presented analysis and results pave the way for other studies. The following directions of future research remain open:

- Further analysis which external or internal factors influence the results of HR estimation, i.e., Image parameters (saturation, hue), type of user’s movements, ROI size, etc.),
- Evaluation of selected algorithms on a larger amount of data,
- Development a metric to detect moments when measurement is correct and reliable,
- Evaluating whether the use of depth and IR channels (provided by the Intel RealSense SR300 camera) as additional sources of pulse signal information increases accuracy.

## 5. Conclusions

Reliable non-contact cardiovascular parameters monitoring can be difficult because many factors can contaminate the pulse signal, e.g. a subject movement and illumination changes. In this article we examined the accuracy of HR estimation for various human activities during typical HCI scenarios (sitting still, reading text, typing text and playing game). We tested three different heart rate estimation algorithms and four signal extraction methods. The results show that the proposed signal extraction method (ExG) provides acceptable results (65% *sRate* for PSD), while being much faster to calculate that the ICA method. We have found that, depending on the scenario being studied, a different combination of signal extraction methods and pulse estimation algorithm ensures optimal heart rate detection results. We also noticed that the choice of signal representation has a greater impact on accuracy than the choice of estimation algorithm.

## Figures and Tables

**Figure 9:**
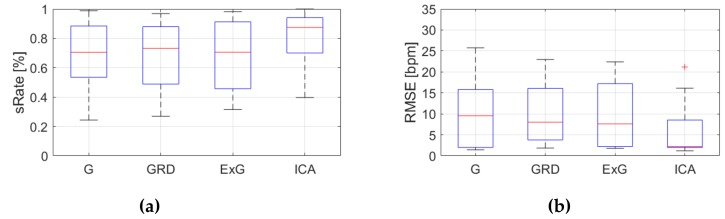
Comparison of signal extraction methods, algorithm No.1 (PSD), part 1: (**a**) box plots for *sRate*; (**b**) box plots for *RMSE*. Blue lines—IQR range, red line—median value.

**Figure 10:**
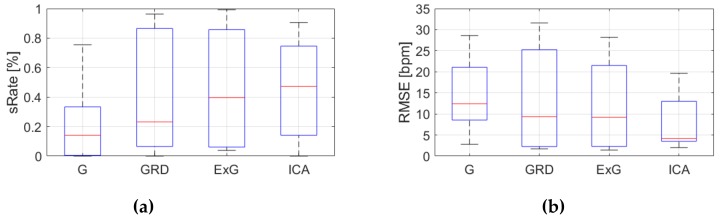
Comparison of signal extraction methods, algorithm No.1 (PSD), part 2: (**a**) box plots for *sRate*; (**b**) box plots for *RMSE*. Blue lines—IQR range, red line—median value.

**Figure 11:**
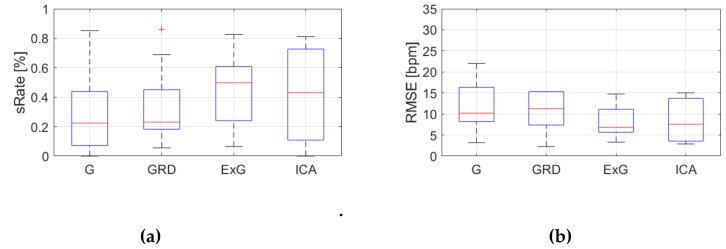
Comparison of signal extraction methods, algorithm No.1 (PSD), part 4: (**a**) box plots for *sRate*; (**b**) box plots for *RMSE*. Blue lines—IQR range, red line—median value.

**Figure 12:**
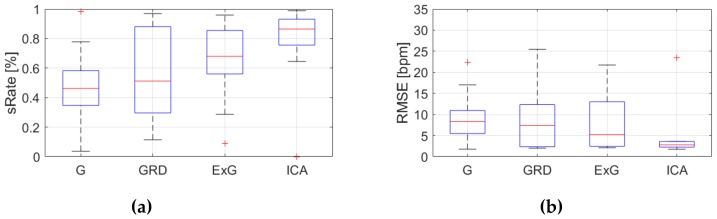
Comparison of signal extraction methods, algorithm No.1 (PSD), part 6: (**a**) box plots for *sRate*; (**b**) box plots for *RMSE*. Blue lines—IQR range, red line—median value.

**Figure 13:**
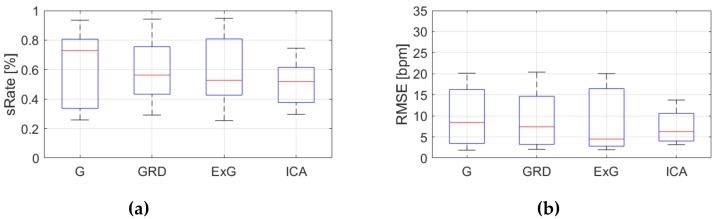
Comparison of signal extraction methods, algorithm No.2 (AR), part 1: (**a**) box plots for *sRate*; (**b**) box plots for *RMSE*. Blue lines—IQR range, red line—median value.

**Figure 14:**
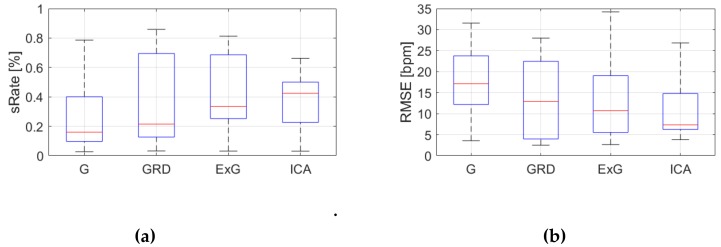
Comparison of signal extraction methods, algorithm No.2 (AR), part 2: (**a**) box plots for *sRate*; (**b**) box plots for *RMSE*. Blue lines—IQR range, red line—median value.

**Figure 15:**
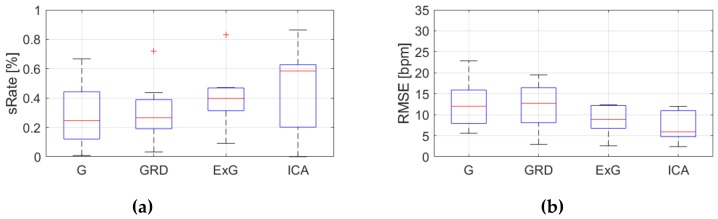
Comparison of signal extraction methods, algorithm No.2 (AR), part 4: (**a**) box plots for *sRate*; (**b**) box plots for *RMSE*. Blue lines—IQR range, red line—median value.

**Figure 16:**
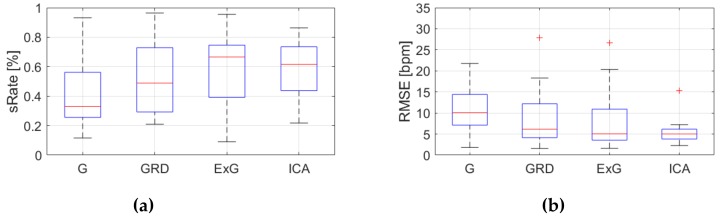
Comparison of signal extraction methods, algorithm No.2 (AR), part 6: (**a**) box plots for *sRate*; (**b**) box plots for *RMSE*. Blue lines—IQR range, red line—median value.

**Figure 17:**
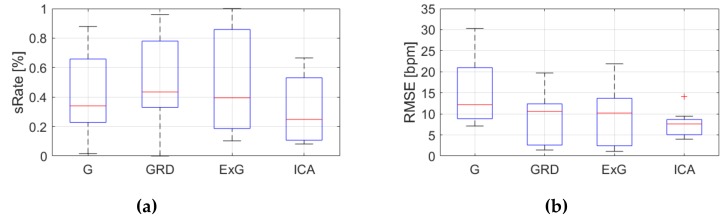
Comparison of signal extraction methods, algorithm No.3 (TIME), part 1: (**a**) box plots for *sRate*; (**b**) box plots for *RMSE*. Blue lines—IQR range, red line—median value.

**Figure 18:**
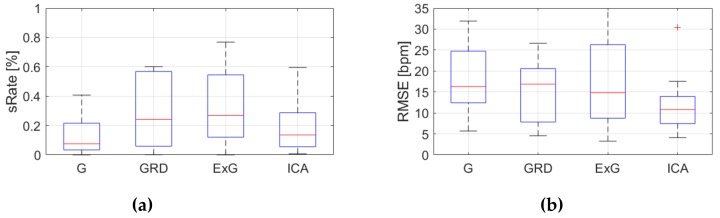
Comparison of signal extraction methods, algorithm No.3 (TIME), part 2: (**a**) box plots for *sRate*; (**b**) box plots for *RMSE*. Blue lines—IQR range, red line—median value.

**Figure 19:**
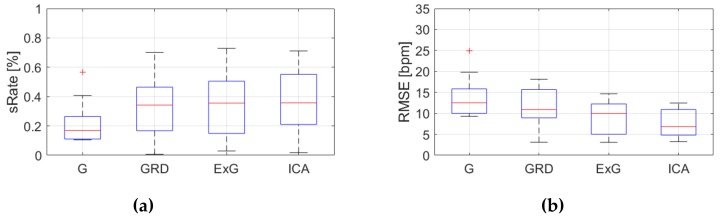
Comparison of signal extraction methods, algorithm No.3 (TIME), part 4: (**a**) box plots for *sRate*; (**b**) box plots for *RMSE*. Blue lines—IQR range, red line—median value.

**Figure 20:**
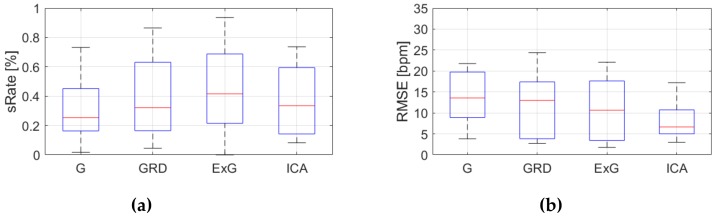
Comparison of signal extraction methods, algorithm No.3 (TIME), part 6: (**a**) box plots for *sRate*; (**b**) box plots for *RMSE*. Blue lines—IQR range, red line—median value.

**Table 1:** Results of the delay estimation for selected algorithms.

Algorithm	t_0_ [s]	t_1_ [s]	t_2_ = t_0_ − t_1_[s]
No.1 (PSD)	13.4	3	10.4
No.2 (AR)	6.6	3	3.6
No.3 (TIME)	5.1	3	2.1

**Table 2:** Recorded video sequences covered by the study.

Video No.	Room Settings	Participant’s Details	Camera Parameters
1	room 1: artificial ceiling fluorescent light + natural light (dusk, medium lighting) from a one window on the left side + light from the one computer screen	participant 1: male, ~34 years old	camera-to-face distance ~50 cm, gain = 128, white balance off
2	room 1: artificial ceiling fluorescent light + natural light (dusk, medium lighting) from a one window on the left side + light from the one computer screen	participant 2: male, ~22 years old	camera-to-face distance ~50 cm, gain = 128, white balance off
3	room 2: daylight (cloudy, poor lighting): a one roof window on the left, and a second window in the back on the right + fluorescent lamps in the back (2 m) + ceiling fluorescent lamps + right-side table lamp + light from two computer screens	participant 3: male, ~44 years old	camera-to-face distance ~50 cm, gain = 128, white balance off
4	room 2: daylight (cloudy, medium lighting): a one roof window on the left, and a second window in the back on the right + fluorescent lamps in the back (2 m) + ceiling fluorescent lamps + light from two computer screens	participant 3: male, ~44 years old	camera-to-face distance ~50 cm, gain = 128, white balance on
5	room 3: daylight (sunny, strong lighting): a one window in the front + light from the one computer screen;	participant 3: male, ~44 years old	camera-to-face distance ~60 cm (computer screen slightly lower – user has to gaze slightly downwards), gain = 100, white balance on
6	room 4: nighttime, artificial light only (ceiling lamps, table lamps, led curtain lamps + light from the one computer screen);	participant 3: male, ~44 years old	camera-to-face distance ~50 cm (computer screen slightly lower – user has to gaze slightly downwards), gain = 128, white balance on
7	room 3: daylight (cloudy, medium lighting): a one window in the front + light from the one computer screen;	participant 4: female, ~42 years old	camera-to-face distance ~60 cm (computer screen slightly lower – user has to gaze slightly downwards), gain = 128, white balance on
8	room 2: daylight (cloudy, poor lighting): a one roof window on the left, and a second window in the back on the right + fluorescent lamps in the back (2 m) + light from two computer screens;	participant 3: male, ~44 years old	camera-to-face distance ~50 cm, gain = 100, white balance off
9	room 5: artificial ceiling fluorescent light + natural light (dusk, medium lighting) from a one window on the right side + right side bulb lamp + light from the one computer screen;	participant 5: male, ~23 years old	camera-to-face distance ~60 cm, gain = 128, white balance on

**Table 3:** The median *sRate* and *RMSE* for selected algorithms and signal extraction methods.

Algorithm	*RMSE* [bpm]				*sRate* [%]			
	G	GRD	ExG	ICA	G	GRD	ExG	ICA
PSD	10.7	13.5	11.1	4.0	47%	49%	65%	78%
AR	15.6	12.3	11.6	5.8	48%	45%	61%	48%
TIME	16.8	13.1	10.9	8.6	35%	43%	45%	26%
average	14.4	13.0	11.2	6.1	43%	46%	57%	51%

## Appendix A

**Table A1:** Duration of the recorded video sequences and selected parts (mm: ss).

Video No.	Entire Video	Part 1	Part 2	Part 4	Part 6
video 1	05:33	01:00	00:25	00:32	01:01
video 2	04:53	00:50	00:19	00:31	01:01
video 3	05:30	00:55	00:24	00:55	01:04
video 4	05:19	00:48	00:18	00:58	01:02
video 5	05:48	01:00	00:26	01:07	01:02
video 6	05:46	01:00	00:28	01:02	01:03
video 7	05:56	00:57	00:32	01:07	01:01
video 8	05:56	01:00	00:20	01:02	01:01
video 9	05:44	01:01	00:34	01:12	00:20

**Table A2:** The average illumination and standard deviation of accelerations for recorded video sequences and selected parts.

Video No.	Entire Video	Part 1	Part 2	Part 4	Part 6	Entire Video	Part 1	Part 2	Part 4	Part 6
	Average Illumination [lux]					std of Accelerations [G]				
video 1	72	78	77	62	69	0.134	0.021	0.010	0.011	0.006
video 2	86	93	91	63	76	0.123	0.007	0.006	0.007	0.007
video 3	40	44	43	39	35	0.112	0.011	0.010	0.009	0.008
video 4	54	60	59	58	46	0.109	0.010	0.008	0.008	0.007
video 5	950	1271	1048	806	816	0.118	0.007	0.008	0.008	0.008
video 6	27	27	30	23	25	0.110	0.007	0.009	0.008	0.007
video 7	152	146	140	113	170	0.125	0.013	0.018	0.011	0.008
video 8	49	57	56	53	38	0.099	0.008	0.007	0.008	0.009
video 9	106	108	107	103	99	0.102	0.009	0.011	0.017	0.011

**Table A3:** Results of HR estimation, algorithm No. 1 (PSD)—comparison of signal extraction methods.

Video No.	*RMSE* [bpm]				*sRate* [%]			
	G	GRD	ExG	ICA	G	GRD	ExG	ICA
video 1	10.7	4.5	3.2	7.8	48%	76%	85%	71%
video 2	16.5	15.2	15.7	14.7	27%	41%	39%	40%
video 3	16.5	13.9	12.9	2.8	47%	49%	57%	83%
video 4	10.1	11.6	7.3	3.2	45%	55%	65%	78%
video 5	3.9	2.2	2.5	2.8	84%	91%	91%	87%
video 6	14.1	13.5	11.1	7.2	43%	42%	65%	62%
video 7	3.5	3.7	3.4	2.2	80%	84%	86%	91%
video 8	6.8	17.9	17.2	4.0	61%	19%	25%	79%
video 9	22.2	36.7	35.8	34.8	11%	16%	15%	12%

**Table A4:** Results of HR estimation, algorithm No. 2 (AR)—comparison of signal extraction methods.

Video No.	*RMSE* [bpm]				*sRate* [%]			
	G	GRD	ExG	ICA	G	GRD	ExG	ICA
video 1	9.5	4.6	3.5	6.3	45%	62%	74%	55%
video 2	17.0	11.1	11.6	13.0	30%	45%	44%	37%
video 3	15.6	15.3	12.2	5.0	48%	45%	61%	68%
video 4	8.6	12.3	7.0	5.7	57%	57%	69%	47%
video 5	3.9	2.6	2.6	3.0	80%	83%	82%	77%
video 6	19.4	17.2	15.4	11.7	23%	27%	46%	35%
video 7	5.4	7.9	6.7	5.8	76%	73%	71%	48%
video 8	15.6	20.6	19.3	4.8	53%	28%	34%	63%
video 9	23.3	35.3	35.5	30.0	10%	13%	12%	9%

**Table A5:** Results of HR estimation, algorithm No. 3 (TIME)—comparison of signal extraction methods.

Video No.	*RMSE* [bpm]				*sRate* [%]			
	G	GRD	ExG	ICA	G	GRD	ExG	ICA
video 1	16.8	4.3	2.8	7.4	49%	70%	83%	51%
video 2	21.3	11.3	10.9	12.2	38%	47%	53%	30%
video 3	20.7	18.4	17.7	6.3	28%	25%	36%	43%
video 4	14.2	13.1	10.4	8.6	35%	43%	45%	21%
video 5	16.7	2.5	2.6	5.5	51%	88%	89%	52%
video 6	20.0	21.4	17.8	12.2	22%	21%	22%	26%
video 7	9.1	9.6	10.9	7.8	50%	47%	48%	18%
video 8	21.4	20.9	21.2	9.7	14%	18%	20%	23%
video 9	16.2	34.0	34.5	29.1	18%	13%	11%	14%

**Table A6:** The Wilcoxon rank sum test results (p-values) for comparing different signal extraction methods.

Comparison	*RMSE* p-Value			*sRate* p-Value		
	PSD	AR	TIME	PSD	AR	TIME
G vs GRD	1.00	0.93	0.49	0.93	1.00	0.93
G vs ExG	0.73	0.49	0.34	0.39	0.55	0.49
G vs ICA	0.14	0.19	**0.01**	0.16	0.86	0.86
GRD vs ExG	0.67	0.67	0.80	0.67	0.60	0.73
GRD vs ICA	0.22	0.30	0.34	0.34	0.86	0.67
ExG vs ICA	0.30	0.39	0.39	0.55	0.60	0.34

**Table A7:** The Wilcoxon rank sum test results (p-values) for comparing different signal extraction methods and activities, algorithm No.1 (PSD).

Comparison	*RMSE* p-Value				*sRate* p-Value			
	Part1	Part2	Part4	Part6	Part1	Part2	Part4	Part6
G vs GRD	0.80	0.49	1.00	0.93	1.00	0.44	0.60	0.80
G vs ExG	0.93	0.39	0.30	0.60	0.93	0.25	0.16	0.22
G vs ICA	0.44	0.09	0.34	**0.05**	0.30	0.16	0.45	**0.04**
GRD vs ExG	0.67	0.86	0.44	1.00	0.80	1.00	0.30	0.86
GRD vs ICA	0.22	0.73	0.39	0.22	0.26	0.75	0.67	0.26
ExG vs ICA	0.30	0.73	0.86	0.19	0.45	0.93	1.00	0.30

**Table A8:** The Wilcoxon rank sum test results (p-values) for comparing different signal extraction methods and activities, algorithm No.2 (AR).

Comparison	*RMSE* p-Value				*sRate* p-Value			
	Part1	Part2	Part4	Part6	Part1	Part2	Part4	Part6
G vs GRD	1.00	0.44	0.80	0.39	0.93	0.55	0.73	0.55
G vs ExG	1.00	0.34	0.39	0.22	1.00	0.19	0.22	0.26
G vs ICA	0.67	0.22	0.09	**0.05**	0.30	0.30	0.26	0.14
GRD vs ExG	0.93	0.73	0.30	0.67	0.86	0.49	0.14	0.60
GRD vs ICA	0.73	0.67	0.14	0.44	0.44	0.73	0.34	0.49
ExG vs ICA	1.00	0.80	0.26	0.73	0.34	0.73	0.60	1.00

**Table A9:** The Wilcoxon rank sum test results (p-values) for comparing different signal extraction methods and activities, algorithm No.3 (TIME).

Comparison	*RMSE* p-Value				*sRate* p-Value			
	Part1	Part2	Part4	Part6	Part1	Part2	Part4	Part6
G vs GRD	0.30	0.49	0.67	0.49	0.49	0.14	0.22	0.55
G vs ExG	0.34	0.80	0.22	0.49	0.60	0.17	0.39	0.34
G vs ICA	**0.01**	0.08	**0.03**	0.06	0.44	0.67	0.22	0.67
GRD vs ExG	0.86	0.80	0.55	0.80	0.86	0.93	1.00	0.73
GRD vs ICA	0.49	0.44	0.22	0.60	0.22	0.49	0.80	0.93
ExG vs ICA	0.44	0.30	0.60	0.86	0.22	0.30	0.86	0.55

**Table A10:** The Wilcoxon rank sum test results (p-values) for comparing different algorithms.

Comparison	*RMSE* p-Value				*sRate* p-Value			
	G	GRD	ExG	ICA	G	GRD	ExG	ICA
PSD vs AR	0.67	0.80	0.93	0.44	0.93	0.80	0.73	0.05
PSD vs TIME	**0.05**	0.73	0.67	0.11	0.26	0.44	0.26	**0.01**
AR vs TIME	0.16	0.80	0.86	0.26	0.22	0.49	0.55	0.06

**Table A11:** The Pearson’s correlation values between the *sRate* and the average scene lighting.

Algorithm	Correlation Value				p-Value			
	G	GRD	ExG	ICA	G	GRD	ExG	ICA
PSD	0.57	0.57	0.45	0.27	0.11	0.11	0.23	0.49
AR	0.56	0.61	0.46	0.47	0.11	0.08	0.22	0.20
TIME	0.50	**0.71**	0.62	0.51	0.17	0.03	0.08	0.16

**Table A12:** The Pearson’s correlation values between the *sRate* and the standard deviation of the accelerations.

Algorithm	Correlation Value				p-Value			
	G	GRD	ExG	ICA	G	GRD	ExG	ICA
PSD	0.27	**0.75**	**0.70**	0.24	0.49	0.02	0.04	0.53
AR	0.32	**0.68**	0.66	0.22	0.41	0.04	0.06	0.56
TIME	**0.87**	**0.72**	**0.78**	0.53	0.00	0.03	0.01	0.14
